# Emerging public health issues due to climate change

**DOI:** 10.4103/0019-5278.44698

**Published:** 2008-12

**Authors:** Harshal T. Pandve

**Affiliations:** Department of Community Medicine, Dr. D. Y. Patil Medical College, Pimpri, Pune - 411 018, India

Dear sir,

Climate change has emerged as one of the most devastating environmental threat. The United Nation's Intergovernmental Panel on Climate Change stated that there is overwhelming evidence that humans are affecting the global climate and highlighted a wide range of implications for human health.[1]

In a news release by the World Health organization on World Health Day (April 7, 2008), it stated that human beings are already exposed to the effects of climate-sensitive diseases and that these diseases today kill millions. They include malnutrition, which causes over 3.5 million deaths per year, diarrhoeal diseases, which kill over 1.8 million, and malaria, which kills almost 1 million. The following examples already provide us with images of the future:

1. European heat wave, 2003: Estimates suggest that approximately 70,000 more people died in that summer than would have been expected.
2. Rift Valley fever in Africa: Major outbreaks are usually associated with rains, which are expected to become more frequent as the climate changes.
3. Hurricane Katrina, 2005: More than 1800 people died and thousands more were displaced. Additionally, health facilities throughout the region were destroyed, critically affecting the health infrastructure.
4. Malaria in the East African highlands: In the last 30 years, warmer temperatures have also created more favorable conditions for mosquito populations in the region and therefore for the transmission of malaria.
5. Epidemics of cholera in Bangladesh: They are closely linked to flooding and unsafe water.

These trends and events cannot be attributed solely to climate change but they are the types of challenges we expect to become more frequent and intense with climate changes. They will further strain health resources that, in many regions, are already under severe stress.[2]

To conclude, climate change already contributes to the global burden of disease and this contribution is expected to grow in the future. Direct as well as indirect effects of climate change are alarming to the public health authorities. It is essential for the health policy planners and administrators to consider climate change as a major public health problem in the near future. The intersectoral coordination is a key in dealing with the climate change. Health planning as well as budget allocations should not be carried out in the traditional manner. Rather, the prime importance should be given to areas where the climate change is affecting the most. Sensitization of general population about climate change and its effects is also an essential aspect.[3] Planning today for the public health issues of tomorrow is the need of the hour.

## References

[1] The IPCC 4TH assessment report.

[2] Climate change will erode foundations of health.

[3] Pandve H (2007). Climate change: Need to sensitize general population. Indian J Occup Environ Med.

